# Nurse Manager Span of Control in Hospital Settings: An Integrative Review

**DOI:** 10.3390/nursrep13040131

**Published:** 2023-11-01

**Authors:** Angel Boned-Galán, Nieves López-Ibort, Ana Gascón-Catalán

**Affiliations:** 1 Hospital Universitario Miguel Servet, 50009 Zaragoza, Spain; acboned@salud.aragon.es; 2 Instituto de Investigación Sanitaria de Aragón (IIS Aragon), 50009 Zaragoza, Spain; nlopezi@salud.aragon.es; 3 Hospital Clínico Universitario Lozano Blesa, 50009 Zaragoza, Spain; 4 Facultad de Ciencias de la Salud, Universidad de Zaragoza, 50009 Zaragoza, Spain

**Keywords:** nurse manager, span of control, leadership, competences, demands, nurse management

## Abstract

Background: Nurse managers face great challenges in responding to organizational demands. There exists a general mismatch between managerial span of control and the demands of the job post, which can lead to overburdening and attrition. The aim of this review was to identify the effects of the nurse manager span of control on hospital organizations. Methods: An integrative literature review was carried out on nurse manager span of control using the Medline, Embase and Web of Science databases. Results: 21 articles were included. Our findings indicate that the span of control of nursing managers influences outcomes in inpatients, professionals, nurse managers, and the organization. Conclusions: The span of control of each nurse manager must be assessed and adjusted to each case. An appropriate span of control will lead to improved outcomes for stakeholders and the organization as a whole. Implications for nursing management: Tools must be developed and implemented to measure and evaluate the span of control of nurse managers. This study was not registered.

## 1. Introduction

Nurse managers (NMs) play an important role, working within their respective care units and departments, which is fundamental to the achievement of the objectives of healthcare organizations [1,2]. The nurse manager position was defined as individuals with direct line responsibility for patient/resident care units, with staff nurses and other care providers reporting directly to them and who have responsibility for hiring and performance management. There is no level of management below them and they may be responsible for managing more than one unit [3]. Over time, the role of NMs has evolved into that of a supervisor of staff performance within care units [4], requiring them to coordinate resources, act as a conduit of information to higher levels of the organization, and communicate the mission, vision, and values of the institution [5]. A core responsibility of NMs is to deploy the best organizational model possible to provide comprehensive care and guidance for the people who depend on them. They are responsible for ensuring the success of their units and the organization as a whole, in addition to safeguarding safety and quality of care [6,7].

In Spain, as in the USA, Canada, Australia, and the United Kingdom, it is increasingly difficult to attract and retain NMs. Several factors may have led to this state of affairs, such as a lack of recognition, low compensation, the increasing complexity of the role, increasing hours, forcing nurses to work overtime and, thus, interfering with personal life, and difficulties in developing a positive supervisor–staff relationship. This set of circumstances raises doubts when choosing to pursue management roles due to the complex demands of the position [8].

A recent review [9] found that factors associated with levels of satisfaction among nursing managers included workload, institutional support, the quality of nurse manager–supervisor relationships, and NM competences and training. Workload analyses must account for the number of staff members reporting to the manager. According to data published in Spain, NMs in public general hospitals manage 27 RNs on average (range, 1 to 105). However, it must be borne in mind that these NMs also manage a similar number of assistant nurses and/or other health professionals (e.g., technicians, physiotherapists), causing this figure to double or even triple [10].

The span of control (SOC), according to its classical definition, refers to the number of employees managed by a superior, measured in full-time equivalents and is also known as the span of management, span of supervision, span of authority, management ratio and span of responsibility. In the context of healthcare, however, this indicator lacks robustness and shows a poor understanding of the complexities of the industry and the aspects that influence the role of NMs [11,12,13]. In the healthcare sector, SOC should not only reflect the direct reports of the NM, but rather should include other aspects such as the purpose, context, resources, and outcomes of the management activity [14].

Generally speaking, SOC choice depends on how the organization is structured with regard to the employee segments and human resources managed by the office of the director of nursing, as well as the number of NMs required by the organization. An increase in the number of management staff involves higher personnel costs and requires appropriate spaces in which managers may carry out their duties [15,16].

However, we must not overlook the trend toward conflating medical and nursing units, causing a single NM to be assigned to each medical unit without taking into account the above-mentioned factors [10].

To date, there has been limited research on the effects of inappropriate or excessive NM SOCs in the healthcare context. This research seeks to fill this gap in the literature by providing a comprehensive analysis of how NM SOCs impact hospital outcomes. Thus, the aim of this review was to identify the effects of the nurse manager’s span of control on hospital organizations.

## 2. Materials and Methods

We chose to perform an integrative literature review, a type of study that includes both empirical and theoretical research [17]. The comprehensive approach used in integrative literature reviews spans problem identification, literature search, data evaluation, data analysis, and presentation of results or findings [18].

We applied the 27-item checklist appearing in the PRISMA 2020 Statement for conducting and publishing systematic reviews.

### 2.1. Search Strategy

The search strategy was used to search the Medline, Embase, and Web of Science databases and was conducted in two stages. The first consisted of a MEDLINE database (via PUBMED) search to identify the keywords used most frequently in papers about the span of control. In the second step, natural words and listed keywords were combined to form the search expression, which was adjusted to the specifics of each database. The search terms were as follows: “nurse administrator”, “nurse manager”, “first-line nurse manager”, “nursing supervisory”, “head nurse”, “nurse management”, “charge nurse”, “span of control”, “span of management”, and “work group size”.

For example, the search strategy used in MEDLINE (via PubMed) was:

(((((((nurse manager[MeSH Terms]) OR (nurse administrator[MeSH Terms]))) OR (“first line nurse manager”)) OR (“nursing supervisor*”)) OR (“head nurse*”)) OR (“nurse management”)) AND (((“span of control”[Title/Abstract]) OR (“span of management”[Title/Abstract])) OR (“work group size”[Title/Abstract])).

### 2.2. Selection Criteria

The inclusion criteria were primary studies and systematic or narrative reviews published in English or Spanish and making reference to the SOC of NMs in hospitals. Due to the scarce research on the subject to date, we chose not to restrict the geographical or temporal scope of the search. The last search took place on 5 May 2022. We excluded those studies that made no reference to the SOC managed by NMs in inpatient health centers as well as research referring exclusively to settings other than healthcare. Opinion articles and letters to the editor were also excluded.

Additionally, we consulted the primary studies identified in the reference lists of the full-text articles selected (cross-references).

### 2.3. Selection Process

Applying the search strategy, we identified a total of 62 articles. After 21 duplicate articles were eliminated, we proceeded to carry out a close reading of the title and abstract of the remaining 37 articles, which resulted in 29 full-text articles included. Following the in-depth reading and review, another 8 articles were excluded because they did not meet the inclusion criteria, leaving 21 for the study: 18 quantitative studies, 2 qualitative studies, and 1 study using mixed methods.

**Figure 1:**
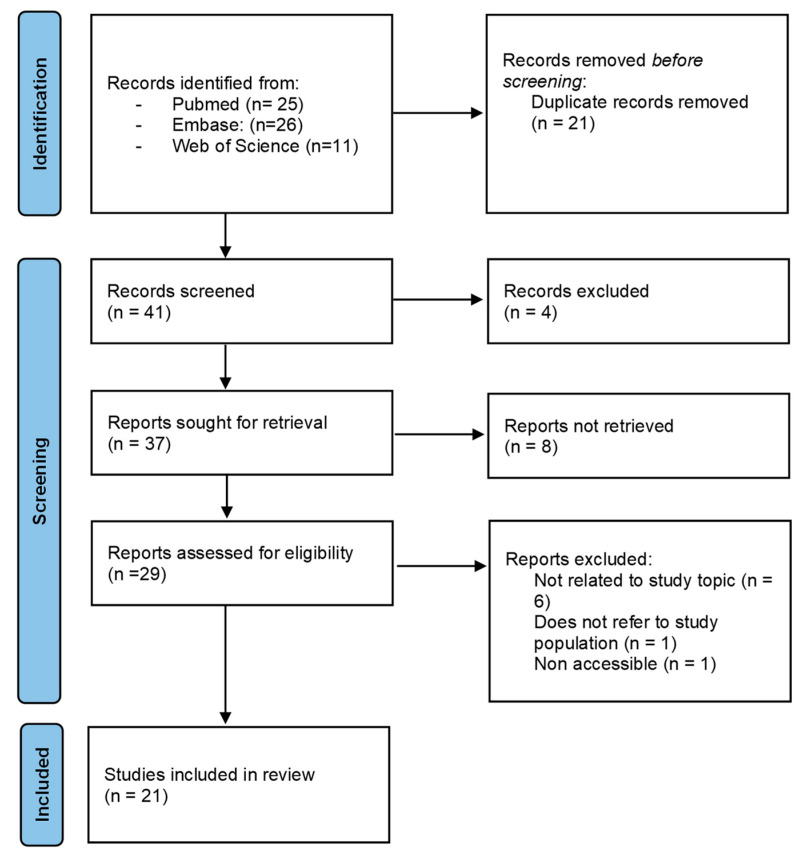
PRISMA flow diagram.

The selection process was carried out with the aid of the PRISMA (Preferred Reporting Items for Systematic Reviews and Meta-analyses) statement. The PRISMA flow diagram for this review is shown in Figure 1.

### 2.4. Quality Assessment

The quality of each publication was assessed using the Johns Hopkins Evidence-Based Practice Model to determine levels of evidence and quality [19].

The level of scientific evidence of research studies enables scale-based evaluation of the scientific rigor of study designs. We used the Hierarchy of Evidence [19] for the present study. This system classifies research articles according to the following criteria: Level I, randomized controlled studies; Level II, quasi-experimental studies; Level III, nonexperimental and qualitative studies; Level IV, opinion of respected authorities and/or nationally recognized expert committees or consensus panels based on scientific evidence; and Level V, experiential and nonresearch evidence. Only three of the studies included in this review were Level II [6,20,21]. The rest were Level III. All were either high or good quality, except one [13], but it was included due to the relevance of the concepts provided.

## 3. Results

**Figure 2:**
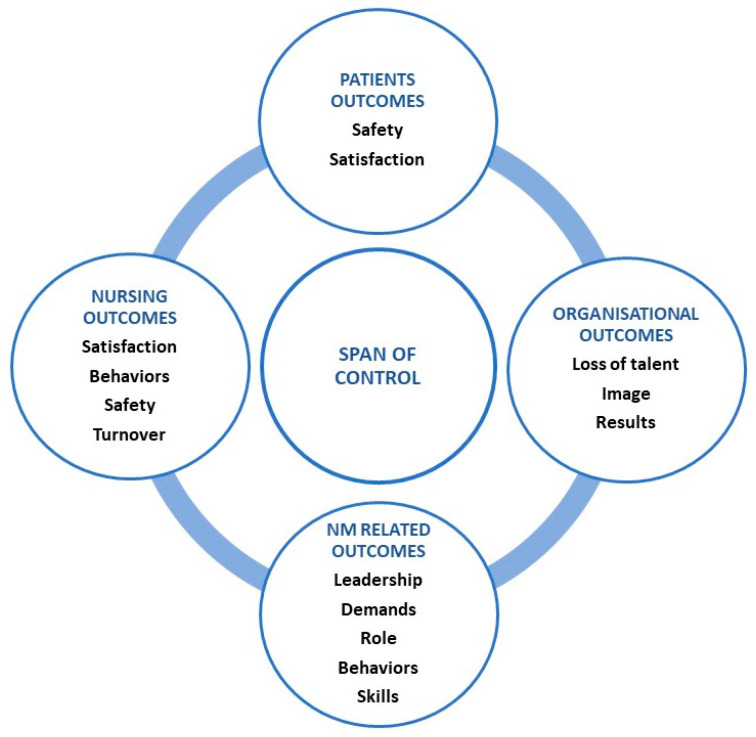
Core concepts of SOC.

The literature review revealed four main areas in which the SOC influences outcomes in healthcare organizations: patients, nursing staff, NMs, and the organization (Figure 2, Table 1 and Table 2).

### 3.1. Patient Outcomes

Three of the 21 articles included in this review focus on the impact of the SOC on patient outcomes, primarily patient safety and satisfaction.

Regarding patient safety, wider SOCs in care units were associated with an increase in medication errors and in the incidence of nosocomial infections [3,32]. Similarly, patient satisfaction was found to decrease in units in which NMs had larger SOCs [25,34].

### 3.2. Nursing Outcomes

Eight articles refer to outcomes related to nursing staff in terms of employee satisfaction, behavior, turnover, and safety.

We found no unanimous results regarding the influence of the SOC on employee satisfaction. While some studies reported that units with larger spans had worse employee satisfaction [3,10,25,33], others did not find evidence of this relationship [23].

Similarly, outcomes concerning behaviors of staff members showed no clear pattern. Studies such as that of Cathcart report worse organizational commitment among workers with a wider SOC. These behaviors improved with an increase in the number of NMs, which caused a fall in manager SOCs [21]. Other authors report no evidence of this relationship regarding organizational commitment [10]. However, these authors observed an inverse relationship between a wider SOC and behaviors such as empowerment, perceived organizational support, and quality of the leader–staff member relationship [10]. We found no association between SOC and citizenship behaviors within the healthcare organizations studied.

In addition to the impact of SOC on managerial behavior, NMs with wide spans were perceived as distant, transmitting no sense of leadership or shared objectives [31].

The articles included contain conflicting results concerning turnover (voluntary resignation or transfer to another unit) or intent to leave (the most significant precursor of turnover behavior) among staff. While some reports found no increase in turnover/intent to leave despite wide SOCs [3,10], authors such as Doran did observe a relationship between the two concepts [34].

Only one study evidenced a relationship between an increased number of workplace accidents and higher SOCs [3].

Despite the negative effects of a wider SOC, larger spans were associated with certain positive outcomes among staff members. Indeed, a wider SOC has been associated with greater flexibility and professional development [31].

### 3.3. Nurse Manager Outcomes

Of the 21 studies included in this review, 16 identified a relationship between SOC and NM leadership, job demands, role, behavior, and skills.

A wide SOC reduces the positive effects of transactional and transformational leadership styles, increasing the negative effects of management by exception and laissez faire leadership styles [25,34]. NMs must change the way they lead [28] to accommodate wider SOCs, as competent leaders capable of effectively managing a wider SOC are better trained in leadership skills and other competences [9,11]. These skills and competences are particularly relevant for less experienced leaders with lower exposure to management responsibilities [29].

Another relevant aspect is the role of the NM, as the demands of the system, which are related to the size of the SOC, are the main drivers of role overload for NMs [3,31]. As described in an improvement project, NMs who receive backing from their institutions report that they are more effective in managing their SOC [30]; the study further found that appropriate technological support and tools should be provided to NMs [9,11,28].

A systematic review of factors that influence NM decisions to remain in their positions indicated that the SOC is a crucial determinant of manager workload and decisions regarding their future in the role [27]. NMs devote a great deal of their time to managing staff and patients and holding meetings [22], which decreases the time available to handle less urgent but more important tasks such as staff professional development, competency assessment, and quality management [22,28]. This increase in demands erodes NM job satisfaction, thereby signaling a need to provide these managers with clinical and administrative support so they may achieve their objectives [23,24,31].

Among the sources consulted, a lower turnover was also observed when NMs had operative and clerical support [30], and these managers were found to have a lower burnout rate [6].

Studies have also explored the influence of a wide SOC on NM behavior and skills, revealing less organizational commitment and empowerment in managers with wide spans [23]. Less experienced NMs, in addition to showing lower organizational commitment, expressed lower perceived organizational support [29].

With regard to the influence of large SOCs on skills, MN emotional intelligence had a lower impact on nurse empowerment [5], and relationships with staff reporting to them and with their own superiors were of lesser quality [10]. Furthermore, a wider SOC was related to problems stemming from miscommunication [31,33].

### 3.4. Organizational Outcomes

Four of the articles selected included this topic. The literature clearly shows that NMs have the widest SOCs and lower support resources within health organizations [13,20].

As mentioned previously, greater workloads resulting from wide SOCs can lead NMs to resign, and as a result, nursing management should ensure that NMs receive the necessary support [27]. The introduction of support measures aimed at reducing the NM SOC can reduce turnover and shorten the time needed to fill vacant positions [30].

## 4. Discussion

Due to the conceptual evolution of SOC over time, it is important to highlight that nowadays, SOC is more than a mere headcount of individuals who report to a given superior. In 1951, Fayol marked a distinction in direct reports as an indicator, taking into account the complexity of the role of these employees and indicating that managerial staff who oversee employees with more multifaceted roles should have fewer employees under their charge [35]. This vision was later taken up and expanded by numerous authors in refining the concept of SOC, particularly in healthcare contexts [11,12,13,24,26,28]. These researchers include aspects that must be subjected to analysis and taken into account when implementing the appropriate measures.

As described in the literature, the following aspects should be considered when determining the SOC of NMs: NM capacity and skills, the degree of interaction or contact between the leader and staff, the scope and complexity of the responsibilities of the position, the number and size of the working groups who report to the NM, the management support available to the NM, the degree of guidance and control required by unit staff, the complexity of the work, and the degree of coordination and planning [14,25,34,36].

Similar work by Morash et al. in creating the Ottawa Hospital Clinical Management SOC Decision-Making Indicators TOH Tool [22] is especially relevant. This instrument assesses eight indicators grouped into three categories (i.e., unit, staff, program), helping to determine the SOC. Several authors in the United States and Canada have used the tool [3,6,30,32], confirming that it is effective for determining the SOC and the support resources that NMs may need.

Although patients are at the center of healthcare and represent its reason for being, few studies have analyzed the influence of an inappropriate SOC on them. Possible causes of a wider SOC leading to an increase in medication errors and increased incidence of nosocomial infections [3,32] may be the lack of proper supervision by the NM, both with respect to the procedures carried out in the unit as well as the behaviors and competences of the staff they supervise. This deficiency in supervision may be due to the need for NMs to focus their time and attention on urgent aspects related to human and material resource management.

The association between lower satisfaction among the direct reports of an NM and wider NM SOCs [25,34] may be explained by the reduced presence and contact of the NM with day-to-day operations in the unit beyond the walls of the manager’s office. Such a separation between leaders and units may prevent them from detecting aspects that should be addressed to improve patient safety and satisfaction.

The high variability in staff satisfaction observed with wide SOCs [3,10,23,25,33] could be related to other factors specific to the units studied, which may mediate the effects of a wide SOC in areas such as employee workload, compensation, staff turnover, or the quality of staff-NM relationship and contact.

We also found no clear pattern regarding the behaviors of nurse managers and the turnover and intent to leave among these individuals [3,10,21]. These differences could be attributable to cultural norms and/or characteristics inherent to the respective health systems, and may be influenced by the way in which these studies were conducted.

The association between an increase in workplace accidents and wider SOCs [3] could be due to insufficient time and clinical support given to NMs, making them unable to monitor procedures or proactively analyze aspects related to the culture of safety. However, given adequate conditions in terms of working environment and professional competence, a wider SOC could be conducive to an environment of growth, flexibility, and professional improvement, especially in the presence of an appropriate leadership style [31].

Leadership is an essential factor in managing units and departments in terms of objective setting and meeting the expectations of staff members and patients. As Doran writes, however, there is no leadership style capable of overcoming the effects of a large SOC [34].

Another relevant aspect is the role of the NM. Much like other tasks linked to healthcare delivery, this managerial role continues to evolve toward greater complexity and increasing demands and responsibilities [37]. This limits interactions between NMs and staff, making it difficult to establish quality relationships between leaders and team members and limiting their availability for staff to assess systems that may improve care quality for patients. There is evidence that manager-staff relationships are less positive with increasing unit sizes [38].

As mentioned above, nursing administrators are in charge of providing NMs with the support they require [27]. In certain situations, failure to provide them with the necessary resources can be construed as a lack of organizational concern for the well-being of these professionals, causing demotivation and burnout. Given the importance of talent management to healthcare organizations, practices that may lead to the loss of NMs are difficult to explain. Over their time in the organization, these leaders acquire know-how that is essential to achieving the objectives of the organization.

Optimizing SOC for NMs holds significant implications for healthcare management. The development of tailored SOC assessment tools is absolutely essential, as these tools empower healthcare organizations to make informed, data-driven decisions that ultimately enhance patient care and staff satisfaction. It is imperative to provide NMs with the necessary support and resources to prevent demotivation and burnout, thereby ensuring the continued delivery of high-quality patient care. In the realm of research, it is crucial to delve into the impact of SOC on patient outcomes and explore the nuanced influence of various leadership styles. These efforts are pivotal in guiding healthcare management towards heightened efficiency and improved care quality.

Improving results is a goal pursued by all healthcare organizations. Based on the evidence reported in the studies analyzed, achieving this objective requires accurate adjustment of the SOC for hospital NMs.

### Limitations

Firstly, it is possible that some studies were inadvertently overlooked, and there may be a selection bias associated with certain constraints, such as language preferences (English and Spanish). This bias could potentially result in the omission of studies conducted in languages other than those considered in this review.

Secondly, it is worth noting that a majority of the included studies originate from the United States and Canada. This geographical bias may impact the generalizability of the study’s conclusions, as the healthcare systems in these regions significantly differ from those in other countries, such as Spain or Slovakia.

Thirdly, the scarcity of research focused on nurses in the field of SOC is a noteworthy limitation. The study of SOC has encountered numerous challenges, including a dearth of empirical studies, difficulties in concept definition, and the emergence of new organizational structures and technologies that hinder the implementation of SOC-related policies [39]. Consequently, it has been necessary to extend the years of search without imposing a publication date limit in order to find an adequate number of studies and address the review’s objective.

## 5. Conclusions

Patient outcomes and the outcomes related to nursing staff, NMs, and organizations will depend heavily on how well-adjusted the SOC is for each manager. This adaptation should be based on an updated concept of SOC that accounts for the various factors described in the literature rather than understandings of SOC as a mere indicator of headcount. Doing so requires tools that empirically, clearly, and periodically measure and evaluate SOC, allowing nursing departments to adapt support for NMs to the specific needs of the care units and departments they manage.

## Figures and Tables

**Table 1:** Results of the literature research.

ID	Title, Author, Year and Country	Study Type	Objetive	Results	Conclusions	Level of Evidence *	Quality Evaluation *
S01	Span of control on nursing inpatient units [13] Pabst 1993. USA.	Descriptive. Case study.	Analyze SOC on several inpatient units.	Determining the optimal SOC requires setting the parameters that go into this metric; these should include efficiency, nurse satisfaction and quality of care. Evaluate nurses’ care delivery, skills, and experience as well as organizational culture.	An investigation of the characteristics of the role and unit is necessary to determine SOC. NMs have a wider SOC than other professional categories in healthcare institutions. Hospital administrators should evaluate nursing SOCs and bring them in line with other employee segments.	III	Low
S02	First-line managers. Measuring their span of control [20] Altaffer 1998. USA.	Descriptive. Survey design.	Examines the SOC and scope of the responsibility of NMs (nurses and non-nurses).	NMs had wider SOCs, fewer assistants, less experience, more training, and a lower salary than non-nursing managers.	NMs have a wider SOC, fewer support staff, lower salaries, and greater reported effectiveness than non-nurses.	III	Good
S03	Span of control matters [21] Cathcart et al. 2004. USA.	Descriptive. Improvement project.	Explore the relationship between SOC and employee engagement.	Worse results were obtained for worker commitment when the SOC was wider. There are 2 significant thresholds: SOCs surpassing 15 and 40 employees. Outcomes related to commitment improved in the 4 units in which a pilot program calling for the addition of an NM was conducted.	A strong relationship between SOC and employee commitment. Employee commitment is at risk in organizations that fail to assess SOC.	II	Good
S04	A span of control tool for clinical managers [22] Morash et al. 2005. Canada.	Mixed methods. Focus group, survey and field test.	Describe the design and implementation of a tool providing guidance on SOC.	Reports an absence of a standardized system for NM SOC determination as well as substantial discrepancies in SOC and range of responsibility among NMs in different units. NMs spent a lot of time coordinating staff and patient care and holding meetings. The managers studied spent little time on workforce development, competence assessment, and quality management.	A questionnaire was designed to aid in SOC decision making, comprising a total of 8 indicators grouped according to the 3 dimensions of staff, program, and unit characteristics: unit complexity, material resource management, direct reports, skills/autonomy (novice professionals), stability (turnover and absenteeism), staff diversity, budget managed.	III	Good
S05	The impact of emotional intelligent leadership on staff nurse empowerment: the moderating effect of span of control [5] Lucas et al. 2008. Canada.	Descriptive. Case study.	To test a model concerning nurse perceptions of emotional intelligence, leadership style, structural empowerment, and NM SOC.	Wider SOCs were associated with a lower impact of the leader’s emotional intelligence on empowerment. (SOC is a significant mediator of empowerment and perception of the emotional intelligence of NMs from the nurses’ point of view.)	NM with high levels of emotional intelligence may be unable to empower their staff if they have a wide SOC. Efforts should be made to ensure that NMs have a reasonable SOC that allows them to develop and use their leadership skills to empower nursing staff so that these professionals may deliver quality care.	III	High
S06	Factors influencing job satisfaction of front-line nurse managers: a systematic review [23] Lee and Cummings. 2008.	Systematic review	Determine the factors that influence NM job satisfaction.	There was no relationship between SOC and manager satisfaction. NM job satisfaction depends on factors such as SOC, organizational support, and empowerment.	NM job satisfaction can be improved by addressing SOC and workload, by adding clerical staff, and by increasing empowerment.	III	High
S07	A Profile of the Structure and Impact of Nursing Management in Canadian Hospitals. [24] Laschinger et al. 2008. Canada.	Descriptive. Case study.	Describe the impact of organizational structure on nurse managers in Canadian hospitals.	Higher SOCs associated with lower job satisfaction. Highlights the importance of redefining SOC beyond number of direct reports.	It is necessary to ensure a manageable SOC so that NMs have sufficient time to support staff, offer guidance, and help them develop. To do so, NMs must be given clinical and clerical support.	III	High
S08	Effects of leadership and span of control on nurses’ job satisfaction and patient satisfaction [25] McCutcheon et al. 2009. Canada.	Descriptive. Survey design.	Examine the relationship between leadership style, job satisfaction, patient satisfaction, and SOC as a mitigating effect.	Units with wide SOCs had worse results in terms of patient satisfaction. Lower effect of transactional and transformational leadership styles on patient satisfaction when SOC is greater. Greater negative impact on employee satisfaction in settings characterized by leadership by exception and laissez-faire leadership.	A wider SOC diminishes the positive effects of transformational and transactional leadership on patient and employee satisfaction outcomes. No leadership style can offset the effects of a wide SOC.	III	High
S09	Managerial span of control: a pilot study comparing departmental complexity and number of direct reports [26] Merrill. 2013. USA.	Descriptive. Case study.	Compare 2 methods of SOC measurement: department complexity vs. number of direct reports.	A moderately positive relationship was observed between the complexity of the unit and number of direct reports.	Use of department complexity, as opposed to the classic model of direct reports, provides a more accurate measure of the scope of NM responsibilities.	III	High
S10	Factors influencing intentions to stay and retention of nurse managers: A systematic review [27] Brown et al. 2013.	Systematic review	Understand the factors influencing NM decisions to remain in the position.	A wide SOC was associated with intent to resign from the post and was also a crucial element determining managerial workload.	Intent to continue as an NM is multifactorial. More senior managers are responsible for supporting NMs as concerns SOC, workload, and work/life balance.	III	High
S11	Exploring managers’ views on span of control: more than a headcount [28] Wong et al. 2014. Canada.	Qualitative research. Focus group	Measure NM perceptions and management of their SOC.	Perception of scope and SOC: Half of the NMs reported having an SOC that was too broad. 5 key factors regarding SOC complexity: demands, role (mainly human resources and distance between units), unit diversity, staff characteristics, and complexity of patients and families. NMs signaled need for clinical support (advanced practice nurses and educators) as well as clerical support. NMs with this support found that it helped them manage their SOC effectively. Need for relational support of equals and superior. Difficulty being proactive and little time to listen to and be with staff. Other aspects: problems with patient family members and patients with different comorbidities requiring involvement of several specialists.	NMs describe system demands as the primary source of work overload in their role. Half of the NMs believe they have an excessively wide SOC and lack the necessary assistance to manage it. Some of the NMs who believe they have a reasonable SOC recognize that they are overloaded and that this excess work prompts them to modify their leadership style. It is necessary to maintain regular contact with NMs to adapt the SOC and support measures according to SOC size as well as other factors that increase the complexity of management.	III	High
S12	Examining the relationships between span of control and manager job and unit performance outcomes [3] Wong et al. 2015. Canada.	Descriptive. Case study.	Examine the characteristics of NMs and SOCs against performance outcomes and the outcomes of the units they manage.	Wide SOC related to high scores on the scale were linked to overload, lower control over the work performed, lower job satisfaction, and worse outcomes in units (i.e., higher rate of medication errors, workplace accidents, nosocomial infections). SOC is not related to staff turnover. The TOH-SOC tool predicted role overload and adverse effects; an inverse relationship was found between job control and employee satisfaction.	SOC exacerbates role overload, increases the likelihood of adverse effects on the unit, and influences job satisfaction and job control. TOH-SOC is an effective tool for determining the support resources that NMs may need to mitigate the effects of a wide SOC.	III	High
S13	The effects of perceived organisational support and span of control on the organisational commitment of novice leaders [29] Havaei et al. 2015. Canada.	Quasi-experimental study	Examine the effects of perceived organizational support, SOC, and leadership on the organizational commitment of novice leaders.	Higher SOC was associated with lower organizational commitment. The negative effects of a broad SOC were reduced in leaders who presented an increased POS (perceived organizational support).	Organizational strategies must be developed to support NMs, and primarily novice managers, to improve organizational commitment.	III	High
S14	Utilizing a scope and span of control tool to measure workload and determine supporting resources for nurse managers [30] Jones et al. 2015. USA.	Descriptive. Improvement project.	Development and implemented a tool, which was used to determine the amount of operational and clerical support for NMs.	Decrease in turnover and time to fill NM vacancies following the implementation of support measures.	Redistribution of operational and administrative resources has a positive impact on NM turnover. Assessment of SOC and NM responsibilities enables determination of the level of operational and administrative support required.	II	Good
S15	Mitigating the Impact of Nurse Manager Large Spans of Control [6] Simpson et al. 2017. USA.	Pre–post study. Improvement project.	Decrease the negative effects of large SOC by providing administrative assistance and individualized transformational leadership development.	Administrative support decreased workloads, freeing up NM time to train staff and connect with patients.	Important to perform annual SOC measurements and provide the necessary support; NMs experience greater satisfaction and less burnout when administrative support and leadership training is provided for them.	II	High
S16	Size does matter–span of control in hospitals [31] Holm-Petersen et al. 2017. Denmark.	Qualitative research. Interviews.	Explore the impact of a wide SOC on staff and their leaders.	NMs seen as a distant and disorganized people who failed to convey a sense of direction and shared goals. Staff felt they were not valued, listened to, or able to access the NM; unaware of what leaders did. Delegating activities to certain nurses created conflicts concerning role and legitimacy with the rest of the staff. Delegation of activities with no follow-up created a feeling of loneliness and frustration The main NM outcomes were the following: Reported sense of inadequacy and frustration. Problems communicating with the group. Difficulty knowing degrees of staff performance. Diversity in cases requiring a higher level of coordination. Delegation of activities with no follow-up. Role overload, work overload, and professional complexity. Perceptions of a large number of meetings and trivial tasks. Positive aspect: wide SOCs provided flexibility and possibilities for staff development.	SOC size influences NM performance and leadership. Additionally, implications for NMs and unit staff perceptions of the work performed.	III	High
S17	Reexamining Nurse Manager Span of Control With a 21st-Century Lens [11] Omery et al. 2019.	Narrative review	Quantitatively and qualitative study and analysis of the consistency of the available evidence on SOC specifically related to NMs.	Leaders with better training and preparation will be able to assume greater SOCs. A wide SOC is incompatible with little support, both in terms of personnel and technology and tools. NMs must have contact with their staff to establish relationships that strengthen empowerment and satisfaction on both sides.	The FTE-based SOC model is outdated. Need for integration of new technologies, use of tools for SOC assessment, and skills development.	III	High
S18	Assessing the Nurse Manager’s Span of Control: A Partnership Between Executive Leadership, Nurse Scientists and Clinicians [32] Cupit et al. 2019. USA.	Descriptive. Improvement project.	Comprehensively assess NM SOCs in the care unit.	44% of NMs fall within the range of excessive SOC and 56% in acceptable range 4 times more medication errors and 3 times higher incidence of nosocomial infections in units with wider SOCs. No differences in patient falls.	When there is excessive nurse manager span of control, patient safety might be compromised particularly by errors and nosocomial infections; no relation with the number of falls recorded.	III	Good
S19	Factors that influence nurse manager job satisfaction: An integrated literature review [9] Keith et al. 2021.	Narrative review	Determine the factors that influence NM job satisfaction.	Reduce SOC as one of the main workloads to improve NM performance and intention to remain in the role. Professional development and adequate resources reflected in greater satisfaction.	NMs carry out their duties better when they have lower workloads, more support, and have participated in competence-development programs. Organizations must systematically evaluate and adjust NM workloads and promote their growth and success	III	High
S20	Communication Skills and Transformative Leadership Style of First-Line Nurse Managers in Relationship to Job Satisfaction of Nurses and Moderators of This Relationship [33] Jankelova and Joniakova. 2021. Slovakia	Descriptive. Case study.	Study the relationship between communication skills and transformational leadership and nurse satisfaction. SOC used as a mitigating factor.	NMs with narrower SOCs felt more secure in their role. Slight moderating effect of SOC on worker satisfaction, transformational leadership, and communication skills.	SOC has a weak moderating effect on communication skills, transformational leadership, and nurse satisfaction.	III	High
S21	Impact of charge nurses’ span of control on the work attitudes of nurses [10] Lopez Ibort et al., 2021. Spain.	Descriptive. Case study.	Establish how SOC affects the organizational behaviors of nurses and the quality of relationships.	Wider SOCs associated with lower scores for empowerment, satisfaction, POS, and quality of leader–nurse relationship. No relationship observed with intent to leave, civic behaviors, or organizational commitment.	SOC size is related to nurse perceptions of organizational behaviors and to the quality of the interpersonal relationships established with the NM.	III	High

NM: Nurse managers; SOC: Span of control. * Johns Hopkins Evidence-Based Practice Model for Nursing and Healthcare Professionals Research Evidence Appraisal Tool.

**Table 2:** List of core concepts of SOC and authors.

Main Area	Concept	Author
Patient outcomes	Safety Satisfaction	S18 S08 S12
Nursing outcomes	Satisfaction Behaviors Safety Turnover	S06 S08 S12 S17 S20 S03 S16 S21 S12 S16 S17
NM-related outcomes	Leadership	S05 S08 S11 S15 S16 S20 S21
	Demand	S04 S06 S10 S11 S12 S14 S16 S17 S19
	Role	S09 S11 S12 S16 S19
	Behaviors	S06 S07 S13 S21
	Skills	S13 S15 S17 S19
Organizational outcomes	Loss of talent Image Results	S10 S14 S02 S01

## Data Availability

All data can be requested from the corresponding author.

## Public Involvement Statement

No public involvement in any aspect of this research.

## Guidelines and Standards Statement

This manuscript was drafted against the guidelines of the Joanna Briggs Institute (JBI) and reported according to the Preferred Reporting Items for Systematic Reviews (PRISMA).
